# The *qTSN4* Effect on Flag Leaf Size, Photosynthesis and Panicle Size, Benefits to Plant Grain Production in Rice, Depending on Light Availability

**DOI:** 10.3389/fpls.2016.00623

**Published:** 2016-05-10

**Authors:** Denis Fabre, Dewi E. Adriani, Michael Dingkuhn, Tsutomu Ishimaru, Bermenito Punzalan, Tanguy Lafarge, Anne Clément-Vidal, Delphine Luquet

**Affiliations:** ^1^Centre de Coopération Internationale en Recherche Agronomique pour le Développement, UMR AGAPMontpellier, France; ^2^Crop and Environment Science Division, International Rice Research InstituteLos Baños, Philippines; ^3^Plant Breeding Genetics and Biotechnology, International Rice Research InstituteLos Baños, Philippines

**Keywords:** *Oryza sativa* L., shade acclimation, quantitative trait locus (QTL), yield potential, panicle size, specific leaf area (SLA)

## Abstract

Increasing rice yield potential is essential to secure world food supply. The quantitative trait locus *qTSN4* was reported to achieve yield increases by enhancing both source and sink capacity. Three greenhouse experiments and one field experiment in the Philippines were conducted to study near-isogenic lines (NILs) in two genetic backgrounds, subjected to treatments with restricted light resources through shading (greenhouse) or population density (field and greenhouse). A consistent promotion of flag leaf width, leaf area and panicle size in terms of spikelet number was observed in the presence of *qTSN4*, regardless of environment. However, grain production per plant was enhanced only in one greenhouse experiment. An in-depth study demonstrated that increased flag leaf size in the presence of *qTSN4* was associated with increased photosynthetic rates, along with lower SLA and greater N content per leaf weight and per area. This was emphasized under low light situation as the *qTSN4*-NILs did not express shade acclimation traits in contrast with the recipient varieties. The authors conclude that *qTSN4* is a promising subject for further physiological studies, particularly under limited radiation. However, the QTL alone may not be a reliable source of increased yield potential because its effects at the plant and population scale are prone to genotype × environment interactions and the increased panicle size is compensated by the adaptive plasticity of other morphological traits.

## Introduction

Rice is a major world food crop (FAOSTAT, 2012) and breeding for improved yield potential is crucial (IRRI, 2010). Despite worldwide production increased due to improved varieties and agronomy, it is stagnating since about 10 years in many regions (Ray et al., 2013). Dingkuhn et al. (2015) recently showed that genetic gains in the yield potential of tropical irrigated rice during the past 20–30 years have been marginal. Actual performances are also threatened by yield instability due to increasingly variable climate and global warming, causing some adaptive abilities the Green Revolution varieties may lack. Today’s high yielding varieties owe their potential to increased harvest index, a result of greater C and N partitioning to the panicle. The greater aggregate panicle sink capacity at population scale, as compared to other sinks in the plant, was enabled by dwarfing. It increased organ number (tillers, leaves, and panicles) while reducing their size, thereby requiring less assimilate investment for maintaining plant standing ability (Peng et al., 2015). Progress in the improvement of the plant’s resource acquisition was also substantial but mainly related to improved light capture through erect canopy architecture and the ability of this architecture to use more N (Peng et al., 2008). However, physiological knowledge on N and C assimilation processes has not been used explicitly in rice breeding, due to their complexity and the difficulty to phenotype them for genetic analyses (Price et al., 2002; Fischer and Edmeades, 2010).

In the humid tropics, grain yield of irrigated rice is often source-limited due to low radiation, particularly during the reproductive and grain filling stages (Liu et al., 2014). Under such conditions, grain yield depends also on remobilization of assimilates from vegetative reserves to harvestable sinks (Jing et al., 2010), in addition to current photosynthesis. Consequently, rice yield potential depends on whole plant source-sink relationships that are determined by many traits (Niinemets, 2014).

Photosynthetic processes constitute the basis of growth and yield where light is limiting. Photosynthetic rate can be limited either by ribulose-1,5-biphosphate (RuBP) carboxylation (Vcmax) or by RuBP regeneration in response to CO_2_ concentration (Jmax), according to Farquhar’s model (Farquhar et al., 1980). Leaf structure and physiology are affected by light level (Estrada-Campuzano et al., 2008; Li et al., 2010; Baldi et al., 2012). Specific leaf area (SLA) increases under low light and the light response of photosynthesis is altered to maximize quantum efficiency at the expense of a lower light-saturated rate (Evans and Poorter, 2001). Sunlit leaves are generally smaller and thicker, with more developed palisade tissue and greater stomatal density compared to shaded leaves. They have less chlorophyll but more electron transfer carriers and RuBP carboxylase/oxygenase (Rubisco) per unit leaf area (Marchiori et al., 2014). Evans and Seemann (1989) also reported that shaded and sunlit leaves differ in N allocation among the functional pools in the leaf to optimize Vcmax and Jmax.

The upper leaves of the rice plant, particularly the flag leaf, provide more than 50% of the assimilates for grain filling (Li et al., 1998). Several studies explored possibilities to genetically improve leaf size and shape, based on both QTL and mutant approaches (Hu et al., 2010; Xiang et al., 2012). Several QTL affecting flag leaf size (Zhang et al., 2015) or panicle sink size were identified (Luo et al., 2013). Among them, *qTSN4* was described as increasing both panicle size (in terms of spikelet number) and flag leaf size (Fujita et al., 2012). This QTL co-localizes with *Nal1* gene, located on chromosome 4, whose loss of function by mutation causes narrow leaves (Qi et al., 2008). The same gene is also involved in the control of photosynthetic rate (Takai et al., 2013).

Fujita et al. (2013) reported a consistent promotion of grains yield by *qTSN4* if introgressed into IR64 rice. However, more research may be needed to evaluate the environment dependency of this effect. Rice yield improvements are either the result of increased biomass production or increased harvest index, or both, regardless of leaf or panicle size. The fact that *qTSN4* is apparently involved both in source traits (flag leaf size, *Nal1* impact on leaf carboxylation rate, Takai et al., 2013) and sink traits (spikelet number per panicle and panicle number, Fujita et al., 2013) suggests that it affects many physiological processes, many of which are prone to environment and trait-by-trait interactions.

This paper aims at providing an eco-physiological analysis of the impact of *qTSN4* on rice plant functioning, with respect to its effect on both C source (flag leaf size, leaf physiology) and sink (panicle size, spikelet number per panicle) processes. For this purpose, two high-yielding *indica* cultivars IR64 and IRRI146 were compared with their respective Near Isogenic Line (NIL) carrying *qTSN4* in four greenhouse and field trials gathering contrasted situations of plant access to light. Flag leaf dimensions, spikelet number per panicle and grain production per plant were fully quantified in these four trials. In one of them (in the greenhouse) genotypes’ photosynthetic characteristics at the leaf level were measured under differential shading conditions, and analyzed together with leaf nitrogen status and non-structural carbohydrate (NSC) levels in the leaf and the stem internode located next to the leaf.

## Materials and Methods

No specific permissions were required for the locations/activities mentioned below because all the experimental sites are in collaborative researches with French Agricultural Research Center for International Development (CIRAD).

### Plant Material

Two high yielding *indica* rice cultivars, IR64, a mega variety, and IRRI 146, second generation NPT (New Plant Type) released in 2007 (Brennan and Malabayas, 2011) designated here as the parents (P) and their respective Near Isogenic Line carrying *qTSN4* (QTL allele increasing spikelet number per panicle, SPN), designated here as NIL (or *qTSN4*), were studied. The NIL were developed by self-pollination of a plant selected from BC3F1 population as described in previous paper (Fujita et al., 2013) and detailed in **Table 1**.

**Table 1:** Plant material description.

Designation	Species	Cross combination	Donor	Category
IR64	Indica	IR64		Recurrent parent recipient
IR64 NIL	Isoline of IR64	IR64/IR68522-10-2-2//3^∗^IR64	IR68	IR64-*qTSN4.4[YP9]*
IRRI 146	Indica	NSIC Rc158		Recurrent parent recipient
IRRI 146 NIL	Isoline of NSIC Rc158	NSIC Rc158/IR65564-2-2-3//3^∗^NSIC Rc158	IR65	NSIC Rc158-*qTSN4.1[YP4]*

### Plant Growth Conditions

#### Multi-trial Analysis

These two pairs of isolines were characterized with respect to flag leaf area (FLA), flag leaf width (FLW), panicle size (in particular spikelet number per panicle, SPN) on the main stem, and plant grain dry weight (PGDW) in a set of one field and three greenhouse experiments in the context of a GRISP (Global Rice Science Partnership) project. In three of the four trials two treatments affecting in some way plant access to light were compared. In one of the four trials only one pair of isolines (IRRI146 genetic background) was addressed. These experiments are summarized in **Table 2**. All experimental sites were used in the context of collaboration between partners owning these sites and CIRAD.

**Table 2:** Description of the trials used for a multi-environment analysis of *qTSN4* effect on plant grain production, flag leaf dimension and spikelet number per panicle on the mains stem.

Trial name	Genotypes	Design	Meteorological conditions
Field IRRI – Dry Season 2014	IR64 + NIL IRRI146 + NIL	Field, four replicates, two treatments (High density: 100 plants/m^2^, low density: 25plants/m^2^)	Average daily temperature: 25.6°C Average daily PPFD: 31.0 mol m^-2.^d^-1^ Total N supply : 160 kg ha^-1^ (i.e., 0.16 g (HD) and 0.64 g (LD) per plant
GH IRRI – Dry Season 2012	IR64 + NIL IRRI146 + NIL	Greenhouse (GH), four replicates, mesh screen at 50% of light attenuation during the crop cycle	Average daily temperature: 25.7°C Average daily PPFD: 15.6 mol m^-2^d^-1^ Total N supply: 0.84 g per 6 L pot
GH CNRS 2013	IR64 + NIL IRRI146 + NIL	GH, three replicates, two treatments (full light and shading at 58% from panicle initiation PI until heading Hd)	Average daily temperature: 27.3°C Average daily PPFD: 10.3 mol m^-2.^d^-1^ (shading); 24.7 mol m^-2.^d^-1^ (control) Total N supply : 0.66 g per 3 L pot
GH IRRI – Wet Season 2014	IRRI146 + NIL	GH, three replicates, two treatments (isolated vs. crowded plants from PI to flowering Flo)	Average daily temperature: 27.7°C Average daily PPFD: 29.8 mol m^-2.^d^-1^ Total N supply: 1.26 g per 6 L pot

#### Greenhouse Detailed Analysis (GH-CNRS 2013)

One of these experiments (GH-CNRS 2013) was dedicated to an in-depth characterization of plant C source-sink relationship underlying *qTSN4* effects. This greenhouse experiment was carried out from May to August 2013 in the National Center for Scientific Research (CNRS, Montpellier, France, 43°38′N, 3°51′E).

The four genotypes, IR64 and IRRI 146 (parents) and their respective NIL (*qTSN4.4-YP9* and *qTSN4.1-YP4*) were grown under natural light in a greenhouse (average of 13 h photoperiod at this season), individually in 3 L pots filled with EGOT 140 substrate (17N-10P-14K, pH = 5). Basal fertilizer was applied using Basacot 6 M at 2 g l^-1^, 11N-9P-19K +2 Mg. Pots were put into four tables with a water layer maintained at about 5 cm depth, each tables containing 104 pots, including border plants, separated of 15 cm each and arranged side by side in a two factors completely randomized design with three replications. The first factor was light treatment including two levels (C: control, with natural daylight, S: stress, under shading), and the second factor was rice genetic background and *qTSN4*. The shading treatment consisting in positioning, all around two dedicated tables, a light-transmitting, (spectrally neutral) gray sun net, from panicle initiation (PI, at 12 leaves appeared on the main stem, 52 to 64 days after transplanting depending on genetic background) until heading stage (33 days after PI). Resulting shading intensity was of 58% compared to the control (0%) under natural light, leading to an average of 10.3 ± 3.4 mol m^-2.^d^-1^ of incident photon flux density (PPFD) in S, and 24.7 ± 7.1 mol m^-2.^d^-1^ in C.

Sun net was installed at 30 cm around (the height was then adjusted with respect to plant growth) the plants to ensure homogeneous microclimate. Microclimate was measured similarly under both treatments. Data-loggers (CR1000 Campbell Scientific) were installed for each treatment to measure air temperature Ta (in average 27.3°C day/23°C night) with a PT1000 probe under fan aspirated shield, air relative humidity RH (in average at 70% day) with HMP45 Vaisala and PPFD with a SKP215 (Skye Instrument quantum sensor, Powys, UK). To minimize side effects, plants on the two external rows of pots on the tables were not used for measurements. The tables were moved every week to avoid bias due to the greenhouse structure.

### Leaf Photosynthesis Measurement

Leaf photosynthesis was measured on the last ligulated leaf, on the main stem at 3 weeks after PI, with a portable photosynthesis system (GFS-3000, Walz, Germany). The measurements were made *in situ* between 9 AM and 1 PM, at saturating PPFD light (1500 μmol m^-2^.s^-1^ of PAR), controlled leaf temperature at 29°C, relative humidity in the cuvette set at 65%, and a constant flow rate through the cuvette of 800 ml min^-1^. The exchanged area of the Walz cuvette corresponding to 8 cm^2^ was fully covered by leaf area. Net photosynthesis-CO_2_ response curves (A/Ci) were constructed over a range of external CO_2_ partial pressure in the following order (400, 300, 200, 100, 50, 400, 600, 800, 1000, 1200, 1400, 1600, and 2000 ppm). At each step, gas exchange variables were recorded after reaching steady-state conditions. Calculations of gas exchange parameters, maximum carboxylation rate (Vcmax), electron transport rate (Jmax), and triose phosphate use (TPU), were computed from each curves using non-linear fitting model developed by Sharkey et al. (2007).

To fit the biochemical Farquhar model of C_3_ photosynthesis to CO_2_ response data, the Rubisco kinetic parameters determined by the temperature response functions were used (Bernacchi et al., 2002).

### Leaf Nitrogen Content and Mass per Area

The leaves used for CO_2_ curves and gas exchange measurements were used for determining, at 3 weeks after PI, N percent of the dry weight (DW; Nm in mg^.^g^-1^ DW of leaf blade), SLA, (cm^2^ g^-1^), leaf mass per area (LMA, the inverse of SLA in g cm^-2^) and accordingly nitrogen concentration on a leaf area basis multiplying Nm by LMA (Na in gN m^-2^). For this purpose, the area of each sample was measured with a leaf area meter (Li-3100 Li-Cor) and then dried in the oven until a constant dry weight was reached (48 h at 70°C). Total leaf nitrogen was analyzed based on Dumas combustion method using a LECO FP-528 Nitrogen analyzer, in CIRAD plant analysis laboratory. Chlorophyll content was also measured on the same leaf using a SPAD-502 (Minolta, Ltd., Japan).

### Sugar Content Analysis

At 3 weeks after PI, NSC (starch, sucrose, glucose, and fructose) content in last ligulated leaf on the main stem and its corresponding internode were characterized using three dedicated plants per genotype. Prior to grind with a ball grinder (Mixer mill MM 200, Retsch, Germany), the samples were frozen in liquid nitrogen. The sugars were extracted three times from 20-mg samples with 1 mL of 80% ethanol for 30 min at 75°C, and then centrifuged for 10 min at 10000 rpm. Soluble sugars (sucrose, glucose, and fructose) were contained in the supernatant and starch in the sediment. The supernatant was filtered in the presence of polyvinyl polypyrrolidone and activated carbon to eliminate pigments and polyphenols. After evaporation of solute with Speedvac (RC 1022 and RCT 90, Jouan SA, Saint Herblain, France), soluble sugars were quantified by high performance ionic chromatography (HPIC, standard Dionex) with pulsated amperometric detection (HPAE-PAD). The sediment was solubilized with 0.02 N NaOH at 90°C for 1 h 30 min and then hydrolyzed with α-amyloglucosidase at 50°C, pH 4.2 for 1 h 30 min. Starch was quantified as described in previous paper (Boehringer, 1984) with 5 μL of hexokinase (glucose-6-phosphate dehydrogenase), followed by spectro-photometry of NADPH at 340 nm (spectrophotometer UV/VIS V-530, Jasco Corporation, Tokyo, Japan).

### Flag Leaf Size, Plant Harvesting, and Related Traits

At heading, FLA, (cm^2^), FLW, (cm) were measured on the main stem of three plants using a leaf area meter (Li-3100 Li-Cor, Lincoln, NE, USA). PGDW (in g), Spikelet Number per panicle (SPN) on the main stem (recoded by P-TRAP software, Al-Tam et al., 2013) were measured at final harvest (physiological maturity of grains). Dry weight was obtained after drying samples at 70°C during 48 h. Plant phenology and development were measured but are presented in another publication (Adriani et al., 2016); accordingly these parameters will not be addressed into detail in the present study.

### Data Analysis

A three-way analysis of variance of treatment, genetic background, QTL and interaction effects on each measured parameter was performed on data from GH-CNRS 2013 and analysis of variance dedicated to split plot design on data from field-IRRI, comparing two treatments and two pairs of isolines) using R (version 3.2.2, R Foundation for Statistical Computing). Where appropriate, mean comparison was performed using Tukey *post hoc* test with the same software.

## Results

### Effect of Treatments on Phenotype

**FIGURE 1:**
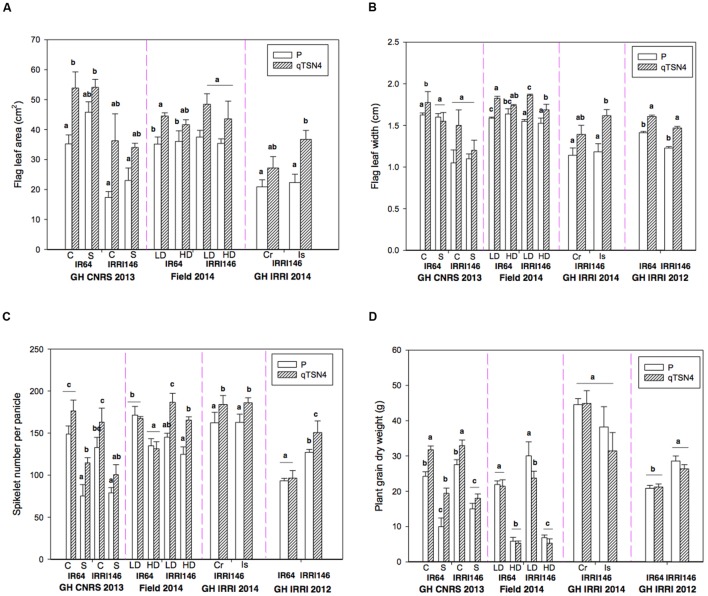
**Morphological characteristics measured at heading and grain physiological maturity in four trials detailed in **Table 2**.** C, control; S, Shading at 58% from panicle initiation to heading; LD, low planting density, HD, high planting density; Cr, Crowded plants from panicle initiation to heading; Is, Isolated plants during this period. Letters indicate the level of significance of *qTSN4* effect between parent and NIL at *p* < 0.05 (Tukey HSD test) in each genetic background in a given experiment. Each bar represents mean ± SE. **(A)** Flag leaf area (FLA; excepted for GH-IRRI 2012). **(B)** Flag leaf width (FLW) on the main stem. **(C)** Spikelet number per panicle on the main stem. **(D)** Plant grain dry weight (PGDW).

According to ANOVA conducted for GH-CNRS 2013 and Field-IRRI 2014 (**Table 3**; **Figure 1**), shading in the GH-CNRS 2013 experiment had no significant effect on FLA and FLW, whereas SPN and PGDW were significantly reduced (*P* < 0.0001). Fourfold increased population in Field-IRRI 2014, which also reduced light availability per plant, did not affect FLA but reduced FLW (*P* < 0.05), in particular for IRRI146 background. The increase in plant density reduces SPN and PGDW (*P* < 0.0001).

**Table 3:** ANOVA *p*-values of flag leaf area (FLA), flag leaf width (FLW), spikelet number per panicle, plant grain dry weight (PGDW), photosynthetic parameters, SLA and N content, and starch content.

Source	Genetic background (G)	QTL	Treatment (T)	G × QTL	G × T	QTL × T
**Flag leaf area**						
GH-CNRS 2013	<0.0001	0.0003	0.2902	0.8243	0.5827	0.1783
Field 2014	0.652	0.001	0.059	0.626	0.937	0.212
**Flag leaf width**						
GH-CNRS 2013	<0.0001	0.066	0.150	0.194	1.000	0.115
Field 2014	0.138	<0.0001	0.035	0.206	0.138	0.012
**Spikelet number per panicle**						
GH-CNRS 2013	0.1897	0.0003	0.2902	0.8243	0.5827	0.1783
Field 2014	0.4095	0.0013	<0.0001	0.0002	0.1395	0.9805
**Plant Grain Dry Weight**						
GH-CNRS 2013	0.089	<0.0001	<0.0001	0.073	0.863	0.922
Field 2014	0.048	0.083	<0.0001	0.155	0.078	0.443
**Photosynthetic parameters in GH-CNRS 2013**						
CO_2_ assimilation	0.0010	<0.0001	0.0004	0.8371	0.1958	0.0023
Vcmax	0.286	<0.0001	0.001	0.921	0.435	0.098
**SLA and N content in GH-CNRS 2013**						
SLA	<0.0001	<0.0001	0.171	0.702	0.907	0.025
Nmass	0.0003	<0.0001	<0.0001	0.1272	0.0569	0.0038
**Starch content in GH-CNRS 2013**						
Leaf starch	0.0025	0.4943	0.0002	0.5985	0.0030	0.4286
Internode starch	0.008	0.028	0.026	0.615	0.946	0.191

In quantitative terms, shading causing 58% PAR reduction caused a 47% reduction in PGDW (GH-CNRS 2013) that could be largely explained by a 41% reduction in SPN. Quadrupling population (Field-IRRI 2014) caused a 76% reduction in PGDW, nearly exactly proportionally to the fraction of PAR resources remaining per plant. The SPN only decreased by 17%, however, and most of the reduction of PGDW was caused by reduced panicle number per plant (Adriani et al., 2016).

In experiment GH-IRRI 2014, the temporary reduction in population during panicle development (increase of spacing among pots) significantly increased FLA and width (**Figures 1A,B**) but did not increase SPN (**Figure 1C**) or PGDW (**Figure 1D**) despite the greater light resources. This surprising result can, however, be explained by the variability observed among plants in this trial, as tiller number was on average higher, at the time when the treatments were established, in plants grown in higher population density compared to those isolated (see Adriani et al., 2016). This initial difference might have hidden the expected, positive effect of population reduction on PGDW.

### Effect of *qTSN4* on Phenotype

Genotype effects were analyzed by ANOVA as three factors, QTL, genetic-background and treatment (**Table 3**) on GH-CNRS 2013 and Field-IRRI 2014. The effects of *qTSN4* on FLA, FLW, SPN, and PGDW were highly significant (*P* < 0.01) in both experiments, except for FLW in GH-CNRS 2013 and PGDW in Field-IRRI 2014 (both *P* = 0.066). Genetic-background affected FLA and FLW significantly (*P* < 0.0001) only in GH-CNRS 2013 and had no effect on SPN and PGDW.

A positive effect of *qTSN4* on FLA was generally observed for both genetic backgrounds (**Figure 1A**). The trend was similar for FLW (**Figure 1B**). A similar positive effect of *qTSN4* was observed on SPN (**Figure 1C**), particularly in IRRI 146 background, whereas in IR64 background the effect was not significant in three of the four trials (GH-CNRS 2013 in C treatment, Field IRRI 2013 and GH IRRI 2012). Consequently, *qTSN4* generally increased flag leaf and panicle size (in terms of spikelet number) but was more consistent in IRRI 146 background.

No consistent effects of *qTSN4* on PGDW were observed. A significantly positive effect (*P* < 0.05) occurred only in GH-CNRS 2013 (except for IRRI 146 under shading) but a negative effect was observed in one case (IRRI 146 field 2014, LD treatment). The effects of *qTSN4* on 1000 grain dry weight was also inconsistent, as a significant reduction was observed in IR64 background in GH-CNRS and field 2014, but a significant increase was observed in IRRI 146 background in GH-CNRS whereas no effect was observed in the field. In fact, the positive effect of the QTL on SPN was compensated by a negative effect on panicle number per plant (as presented in Adriani et al., 2016), resulting in the variable (positive or negative) effects on PGDW.

Across all experiments and treatments, *qTSN4* increased FLA by 38% FLW by 17%, SPN by 22%, PGDW by 7%, and it reduced panicle number per plant by 10% (as presented in Adriani et al., 2016). The most consistent effect of *qTSN4* was on FLW, in terms of orientation of the effect and the number of environments where it was significant. In this study, we did not observe any visible differences among genotypes regarding leaf angle. The 17% increase translated into a greater absolute effect on a leaf area basis (38%) because leaves for *qTSN4* were both wider and longer.

Regarding plant development and phenology, it was observed that *qTSN4* reduced the rate of tillering early, i.e., before PI for both genetic backgrounds across environment (as presented in Adriani et al., 2016), which was not reported in previous studies (Fujita et al., 2012, 2013).

### *qTSN4* Interactions with Treatments and Genetic Background

No statistically significant *qTSN4* by treatment interactions were observed for FLA, FLW, SPN, or PGDW in the GH-CNRS 2013 and Field-IRRI 2014 experiments, except for FLW (*P* = 0.012) observed in the field experiment (**Table 3**). However, the QTL effect on FLA was on average greater under control conditions (+81%) than shading (+33%) for GH-CNRS 2013, and it was also greater for control conditions (+28%) than high population (+19%) in Field-IRRI 2014 (**Figure 1A**). The same pattern was also observed for FLW (**Figure 1B**).

Interactions between *qTSN4* and genetic background were generally not significant except for SPN observed in Field-IRRI 2014 (*P* < 0.001, **Table 3**). However, *qTSN4* effects on FLA were greater in IRRI 146 (+78%) than in IR64 (+36%) background at GH-CNRS 2013. The same trend was observed at Field-IRRI 2014 but effects were smaller (26 and 21%, respectively). No such trends of treatment and background dependency of QTL effects were observed for SPN and PGDW.

### Photosynthesis

**FIGURE 2:**
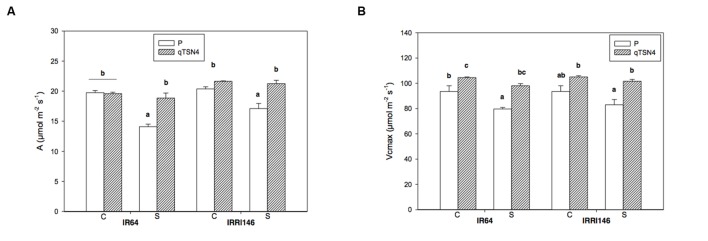
**Physiological leaf characteristics measured at 3 weeks after panicle initiation in greenhouse experiment (GH-CNRS 2013) with two light treatments.** C, Control; S, Shading. Different letters indicate significant difference at *p* < 0.05 (Tukey HSD test) among values in each genetic background in a given experiment. Each bar represents mean ± SE (*n* = 3). **(A)** Photosynthetic rate. **(B)** Maximum carboxylation rate (Vcmax).

Net CO_2_ assimilation rate at ambient CO_2_ concentration of 400 ppm (*A*_400_) was measured only in GH CNRS 2013 trial and results are presented in **Figure 2A**. Shading significantly (*P* < 0.01) reduced *A*_400_ under PAR saturation for IR64 (-29%) and IRRI 146 (-16%) parents whereas the respective *qTSN4* NILs were unaffected. Consequently, there was a strong *qTSN4* by shading interaction (*P* < 0.01) in addition to the significant *qTSN4* effect (*P* < 0.0001) and shading effect (*P* < 0.001) alone (**Table 3**). Similar observations were made for Vcmax, but in contrast to *A*_400_, *qTSN4* had a promoting effect under both control and shaded conditions (**Figure 2B**). As a result, only *qTSN4* and shading effects on Vcmax were significant whereas the interaction was not (**Table 3**).

We measured *A*_400_ only at light saturation and consequently, quantum efficiency (QE; initial slope of light response curve) is not known.

The Jmax/Vcmax ratio, indicative of resource allocation between the two photosynthetic cycles, electron transport and the Calvin Benson cycle, was not statistically different among genotypes.

### Specific Leaf Area and Nitrogen Content

**FIGURE 3:**
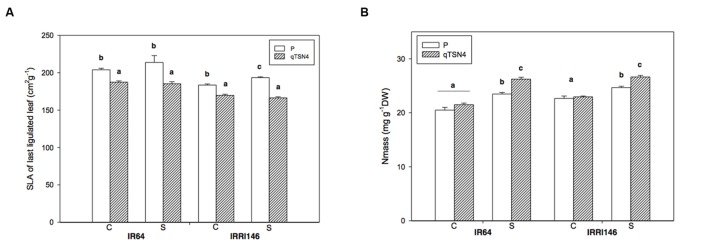
**Leaf anatomical characteristics and N status measured at 3 weeks after panicle initiation in greenhouse experiment (GH-CNRS 2013) with two light treatments.** C, Control; S, Shading. Different letters indicate significant difference at *p* < 0.05 (Tukey HSD test) among values in each genetic background in a given experiment. Each bar represents mean ± SE (*n* = 3). **(A)** Specific leaf area (SLA). **(B)** Dry weight based leaf nitrogen content (Nm).

Specific leaf area (SLA) was measured on the leaf used for photosynthesis measurement at 3 weeks after PI in GH-CNRS 2013 trial. A significant, negative QTL effect (<0.0001) was observed, whereas the shade treatment had no effect (**Table 3**; **Figure 3A**). Consequently, the presence of *qTSN4* made leaves not only larger but also thicker. In both genetic backgrounds, the *qTSN4* effect on SLA was greater for shade treatment than control (**Figure 3A**), resulting in a significant (*P* = 0.025) *qTSN4* by shading interaction (**Table 3**). There was also a significant (*P* < 0.0001) genetic-background effect on SLA, IRRI 146 having lower SLA than IR64. However, the phenotypic expression of *qTSN4* did not interact with the background.

On the same leaves, dry weight-based leaf nitrogen concentration (Nm) was also measured. Shading significantly increased Nm (*P* < 0.0001; +9% for the parents and +16% for the *qTSN4* NILs; **Figure 3B**). No *qTSN4* effect on Nm occurred under control conditions but the QTL had a positive effect under shading in both genetic backgrounds (*P* < 0.0001; 8% in IRRI 146 and 12% in IR64 backgrounds; **Figure 3B**). The patterns observed for Nm were similar to those for *A*_400_ (**Figure 2A**). They were also similar to the reciprocal of SLA, indicating that *qTSN4* induced thicker leaves (lower SLA) with greater N concentration and higher *A*_400_.

**FIGURE 4:**
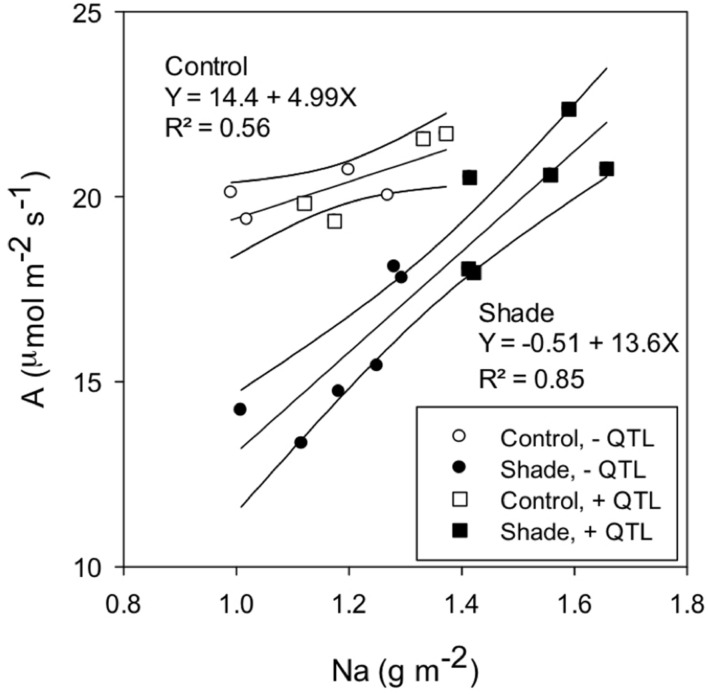
**Relationship between photosynthetic rate at PAR saturation (A) and the leaf area N content (Na) for control and shade-acclimated plants in experiment GH-CNRS 2013.** Presence or absence of *qTSN4* is indicated by ±QTL.

Positive correlations were observed between *A*_400_ and the area-based leaf nitrogen content (Na), which is equal to Nm SLA^-1^ (**Figure 4**). However, the *A*_400_ vs. Na slope was greater for the shade than the control treatment. The presence of *qTSN4* did not affect the response of *A*_400_ to Na, indicating that greater *A*_400_ caused by *qTSN4* under shade was related to greater Na.

### Leaf and Internode Starch Content

**FIGURE 5:**
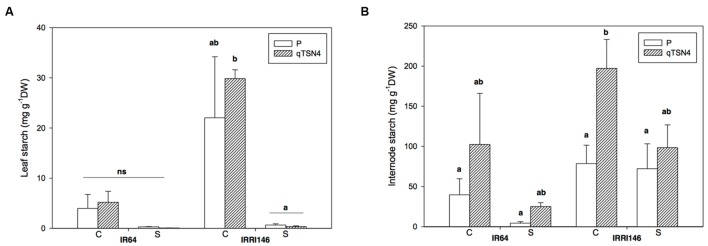
**Starch content measured at 3 weeks after panicle initiation in greenhouse experiment (GH-CNRS 2013) with two light treatments.** C, Control; S, Shading. Different letters indicate significant difference at *p* < 0.05 (Tukey HSD test) among values in each genetic background in a given experiment. Each bar represents mean ± SE (*n* = 5). **(A)** Leaf starch. **(B)** Internode starch.

Starch contents were analyzed 3 weeks after PI on the last ligulated leaf on the main stem, and its related top internode (**Figures 5A,B**; **Table 3**). Significant (*P* < 0.05) effects of shading, *qTNS4* and genetic background were observed for internode starch content. Namely, shading reduced it, *qTNS4* increased it, and between the backgrounds, IRRI 146 had greater concentrations than IR64. No significant factor interactions were observed.

In leaf blades, shading and genetic background both had significant (*P* < 0.01; **Table 3**) effects on starch concentration, whereby IRRI 146 had higher values than IR64 and shading made starch virtually disappear (**Figure 5A**). There was a strong treatment by genetic-background interaction on leaf starch (*P* < 0.01).

Regarding soluble sugar concentrations (data not presented), no *qTNS4* effect was observed on hexose or sucrose content in leaves and internodes for IR64 background. In IRRI 146 background, *qTNS4* reduced soluble sugar concentrations in internodes (*P* < 0.05). Leaf soluble sugar content was in general decreased by shading.

## Discussion

This study analyzed the phenotypic expression of *qTSN4* in two genetic backgrounds and for several environments and experimental treatments affecting light resources. We thereby focused mainly on the organ level (leaf and inflorescence traits) without trying to evaluate the QTL agronomically.

The most reproducible effect across experiments and environments was the increase of flag leaf size. Flag leaf size is known to be related to panicle size (Dingkuhn et al., 2015) and thus is of interest to breeders, for example in the quest in China to develop higher yielding ideotypes using molecular markers (Zhang et al., 2015). Although the name of *qTSN4* stands for Total Spikelet Number (at the scale of the panicle), the gene likely to cause its effect, *Nal1*, causes narrow leaves when dysfunctional, and thus is actually a large-leaf gene (Qi et al., 2008).

### Stability of QTL Phenotypes

*qTSN4* has the particularity to be involved both in the size of sink (panicle size, or spikelet number per panicle, SPN) and source (FLW and area) organs (Fujita et al., 2012). In addition, this QTL co-localizes with *Nal1* gene, a mutation that was reported to affect the growth in leaf width, carboxylation activity and photosynthetic rate (Qi et al., 2008). A positive effect of the QTL under field conditions was reported by Fujita et al. (2012). Our 2013 greenhouse study appeared to confirm this but the 2014 field study could not confirm such grain production gains. These contradictory results may be related to variable trade-offs between the sink size of the individual panicle, which is increased by *qTSN4*, and panicle number per plant or field area, which usually is smaller when panicles are large. Phenotypic compensations between size and number of organs such as leaves, tillers, or panicles are common in rice, making it difficult to increase yield potential with one trait alone (Dingkuhn et al., 2015). Even if both source and sink capacity are increased at the scale of a tiller or panicle, as observed for *qTSN4*, yield potential is not necessarily increased at field scale. This suggests that the phenotype of *qTSN4* is plastic, or prone to QTLxE interactions. As this QTL is involved in morphological traits that are known to be phenotypically plastic, such interactions may depend on environmental and crop management effects on the source-sink balance. In this study, this hypothesis was addressed by studying the variation of *qTSN4* effects on source organs (flag leaf morphology and photosynthetic rate) and sink organs (panicle size and number), through various experiments providing differential light resources. Morphological effects of *qTSN4* on the flag leaf (increased area, width, thickness in terms of SLA) and the panicle (larger spikelet number) were highly significant and expressed in the greenhouse, in the field, and under shading and increased plant population treatments. Although QTL by treatment interactions were mostly not significant for the morphological traits, QTL effects varied in magnitude. For the leaf size, which had less phenotypic plasticity than panicle size, *qTSN4* had a greater effect under the more favorable control conditions. This trend was not observed for panicle size, in terms of spikelet number. Dingkuhn et al. (2015) reported that flag leaf and panicle size are positively correlated among rice genotypes, but panicle size is extremely plastic in variable environments whereas leaf size is comparatively stable. We thus hypothesize that *qTSN4* has a constitutive, stable effect on FLA whereas its effect on panicle size, although generally positive, is more prone to physiological interactions with other traits and with environment. The observed interactions of *qTSN4* with the genetic background support this hypothesis. The QTL promoted FLA in all four experiments, all environments and both backgrounds, but it promoted panicle size in all environments only for IRRI 146. In IR64 background, *qTSN4* did not promote panicle size in the field experiments regardless of the treatment, and it also did not promote it in the 2012 IRRI greenhouse experiment.

### Does *qTSN4* Control Shade Adaptation?

Plant species vary in their capacity to modify leaf morphology, leaf physiology, and canopy structure in response to low light (Sack et al., 2006; Rozendaal et al., 2013), which can be interpreted as adaptive phenotypic plasticity (Bradshaw, 2006). One such response to low light is the increase in SLA, which increases light capture by spreading out leaf biomass on a greater surface and thereby increasing plant relative growth rate when light interception is limiting growth (Evans and Poorter, 2001). Plasticity of SLA is under genetic and physiological control (Valladares et al., 2000). In our greenhouse study, SLA increased in response to low light in the IRRI 146 and IR64 background varieties, as commonly observed in rice and other species (Terashima and Hikosaka, 1995; Evans and Poorter, 2001; Lafarge et al., 2010). Interestingly, SLA of the *qTSN4* NILs did not change in response to shading. Photosynthetic rates measured under light saturation were maintained under shading for the *qTSN4* NILs while they were reduced for the parents. This might indicate that *qTSN4* suppressed the expression of shade acclimation processes.

Shade acclimation, beyond the structural changes responsible for increased SLA, commonly changes the response pattern of photosynthetic rate to PAR. This results in increased QE, favoring photosynthetic rate at low PAR, at the expense of maximal rates because saturation happens at lower PAR (Givnish, 1988). We did observe lower rates at light saturation but PAR response curves were not available. Unlike species of forest ecosystems (Valladares et al., 2000; Yamashita et al., 2000; Valladares, 2003), fast-growing annual grasses such as cereal crops have limited capacity to develop typical shade leaves. However, leaves of barley developing under shade develop acclimation characteristics such as greater SLA and individual leaf area, as well as lower maximal photosynthetic rates (Zivcak et al., 2014).

The present study showed that Nm was higher under shading compared to control treatment, and that this was more pronounced for the *qTSN4*-NILs (**Figure 3B**). Given the finite soil and N resources in the pots, it is likely that growth reductions caused by shading increased Nm because absorbed N was less diluted. It is common that Nm is greater in shade than sun leaves, whereas Na is not necessarily increased because of higher SLA (An and Shangguan, 2008). Increased *A*_400_ in the presence of *qTSN4* was probably a result of thicker leaves (lower SLA) while having greater Nm, both contributing to increased Na. The promoting effects of *qTSN4* on *A*_400_ can thus be explained by morphological and N partitioning effects causing greater photosynthetic capacity. This effect was also demonstrated for *Oryza sativa* and *O. glaberrima* parents and their crosses that genotypic differences in SLA are negatively correlated with leaf chlorophyll content and photosynthetic rates (Dingkuhn et al., 1998). It thereby apparently disabled or counteracted the shade adaptations observed in the recurrent parent, in terms of SLA and *A*_400_. The measurements did not allow evaluating any more direct effects of *qTSN4* on photosynthetic processes, e.g., carboxylation rate as reported by (Takai et al., 2013) for the *Nal1* gene associated with *qTSN4*, because the observed increase of Vcmax might be a direct effect of increased Na and thus, Rubisco.

### Role of Starch in the Sequence of *qTNS4* Effects on C Source-Sink Balance

Carbohydrate reserves in vegetative tissues serve as a buffer for the short term (diurnal; in leaf blades) or longer periods (stems; Gibon et al., 2009; Scofield et al., 2009; Ludewig and Flügge, 2013). Starch storage, particularly in leaves, is a key transient assimilate sink for maintaining both plant growth and photosynthesis, by stabilizing the source-sink balance (Gibon et al., 2009; Sulpice et al., 2009). Stored assimilate in internodes can be remobilized if assimilate shortfalls occur during grain filling (Ruuska et al., 2006), for example under N-limited conditions (Pan et al., 2011). Pre-anthesis reserves in stems are associated with enhanced spikelet development, resulting in increased sink strength of spikelets on inferior positions on the panicle (Fu et al., 2011). *qTSN4* promoted accumulation of starch both under control and shade conditions in internodes but not in leaf blades. This may be a result of the larger leaf size and higher Na induced by the QTL, enabling greater assimilate production per culm. The larger pool of starch stored in the stem by the *qTSN4*-NILs in both genetic backgrounds and both treatments in GH-CNRS might explain the larger panicle sink.

Higher photosynthetic rates in flag leaves can be either the cause of increased stem starch accumulation (by spill-over) or its result (because a strong storage sink in stems would enhance leaf sugar export), thereby stimulating the Calvin cycle (Paul and Foyer, 2001; Kaschuk et al., 2009). The colocalization of *qTSN4* with the *Nal1* gene, associated with pleiotropic effects on leaf anatomy and photosynthesis (Takai et al., 2013), suggests that the QTL affected photosynthesis whereas stem starch storage was a secondary consequence.

### How Promising Is *qTSN4* for Rice Crop Improvement ?

Four independent experiments and various treatments restricting radiation per plant (shading and population density) confirmed a consistent promotion of FLW, leaf area and panicle size in terms of spikelet number. However, only in one greenhouse experiment, grain yield per plant was enhanced. The in-depth study (GH-CNRS 2013) demonstrated increased photosynthetic rates in the presence of *qTSN4*, along with lower SLA and greater Nm and Na, while it appeared that in *qTSN4*-plants the typical shade acclimation traits observed in the recurrent parents were not expressed. This makes *qTSN4* a promising subject for further physiological studies, particularly under limited radiation and N availability, but the QTL alone is not necessarily a sufficient source of improved yield potential because single-panicle or single-culm traits are usually compensated by the adaptive plasticity of other morphological traits. The leaf and panicle traits conveyed by *qTSN4*, although much sought-after by breeders, thus need to be combined with other traits to achieve consistent yield increases at field scale. No observations on the root systems were made in this study. This may warrant more research because it was reported that *qTSN4* increases root weight (Fujita et al., 2013).

## Conclusion

The present study confirmed that *qTSN4* is directly or indirectly involved in both C source and sink processes during the early reproductive phase of rice. With this respect, its effect on plant grain production was shown to depend on the environment, in particular light level, and the way it affects C source-sink relationships, namely leaf size, photosynthetic rate, starch metabolism, and panicle size and number. These results need, however, to be comforted across a wider range of cropping conditions conferring various levels of competition for light. The relation between *qTSN4* effect and N use efficiency under low light situation suggests also that future trials aimed to confirm the E dependent effect of *qTSN4* should deal with different levels of light and N. The present findings are, however, original and provide further insight on the cropping situations where *qTSN4* can be interesting for breeding.

## Author Contributions

DL, TL, and MD participated in the designed of the study. DF, DA, BP, TL, DL, and MD participated in performing the research. TI provided seed materials for the experiments. DF, DA, DL, and MD participated in analyze data and wrote the paper. All authors read and approved the final manuscript. AC-V participated in performing sugar content analysis.

## Conflict of Interest Statement

The authors declare that the research was conducted in the absence of any commercial or financial relationships that could be construed as a potential conflict of interest.
